# Density functional theory study of Au-fcc/Ge and Au-hcp/Ge interfaces

**DOI:** 10.3762/bjnano.14.90

**Published:** 2023-11-15

**Authors:** Olga Sikora, Małgorzata Sternik, Benedykt R Jany, Franciszek Krok, Przemysław Piekarz, Andrzej M Oleś

**Affiliations:** 1 Faculty of Materials Engineering and Physics, Cracow University of Technology, Podchorążych 1, PL-30084 Kraków, Poland; 2 Institute of Nuclear Physics, Polish Academy of Sciences, W. E. Radzikowskiego 152, PL-31342 Kraków, Poland; 3 Marian Smoluchowski Institute of Physics, Faculty of Physics, Astronomy and Applied Computer Science, Jagiellonian University, Łojasiewicza 11, 30348 Krakow, Poland; 4 Institute of Theoretical Physics, Jagiellonian University, Prof. Stanisława Łojasiewicza 11, PL-30348 Kraków, Poland

**Keywords:** Au/Ge heterostructures, density functional theory, electronic structure, hexagonal gold, interface energy

## Abstract

In recent years, nanostructures with hexagonal polytypes of gold have been synthesised, opening new possibilities in nanoscience and nanotechnology. As bulk gold crystallizes in the fcc phase, surface effects can play an important role in stabilizing hexagonal gold nanostructures. Here, we investigate several heterostructures with Ge substrates, including the fcc and hcp phases of gold that have been observed experimentally. We determine and discuss their interfacial energies and optimized atomic arrangements, comparing the theory results with available experimental data. Our DFT calculations for the Au-fcc(011)/Ge(001) junction show how the presence of defects in the interface layer can help to stabilize the atomic pattern, consistent with microscopic images. Although the Au-hcp/Ge interface is characterized by a similar interface energy, it reveals large atomic displacements due to significant mismatch. Finally, analyzing the electronic properties, we demonstrate that Au/Ge systems have metallic character, but covalent-like bonding states between interfacial Ge and Au atoms are also present.

## Introduction

Heterophase interfaces are responsible for unique properties of many advanced devices designed for electronics and other applications [1]. Understanding the formation and energetics of interfaces is highly important for the nucleation of new crystalline phases on a specific substrate [2–3]. The structure of a heterophase can be studied using advanced atomic-resolution experiments, such as high-resolution electron microscopy [4], high-resolution secondary-electron microscopy [5], scanning transmission electron microscopy [6–7] or scanning tunnelling microscopy. However, experimental data provide only partial insight into the formation of interfaces, and the interpretation of microscopic images is often ambiguous. Various simulation techniques can be used to resolve the uncertainties and reveal mechanisms stabilizing the observed interfaces. Deeper knowledge of surfaces and interfaces in heterostructures can play a crucial role in developing new methods for synthesizing such materials and in expanding their possible applications in nanoscience and nanotechnology. As a result of advances in computational methods and the increasing computer power, the atomic structure and specific properties of various interfaces have been successfully studied using first principle calculations [8–13].

Up to very high pressures, gold crystallizes in the fcc phase [14] with the ABC repeating pattern of hexagonal planes in the [111] direction. Recently, nanostructures including different polytypes of gold have been observed in various experiments and have gained interest regarding potential applications. For example, the interface regions of polytypic gold nanorods were found to be highly active in catalysis [15]. The hexagonal phases seen in experiments are characterized by ABAB (hcp) or ABAC (dhcp) stacking patterns. The hcp surface has been observed experimentally in nanowires [16] and ultrathin sheets on graphene oxide [17]. Nanoribbons with metastable dhcp structure have been also reported [18–19] and were used to grow the dhcp forms of several other metals. It is worth mentioning that also stable hexagonal silver dhcp nanostructures have been synthesized [20–21].

Nanostructures of hcp gold were found after growing Ge nanowires with Au as catalyst [22]. A possible mechanism responsible for the formation of hcp gold has been suggested, involving a hexagonal AuGe β phase present during the intermediate stages of growth. Interestingly, while the fcc crystallites were randomly oriented with respect to the Ge substrate, the hcp nanostructures were typically found with (001) planes at 60–65° to the (111) Ge planes [22]. In a recent experiment, stable hcp nanoislands were obtained under controlled annealing conditions on the germanium substrate [23]. After initial crystallization of the fcc gold phase, the hcp phase grows from the eutectic Au/Ge liquid. The atomically resolved STEM-HAADF measurements as well as electron backscatter diffraction results show that the Au-fcc borders the Ge(001) surface, whereas a preferred hcp crystal orientation is when the Au(010) plane, or Au(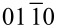) in the Miller–Bravais notation, is parallel to the Ge(111) plane. An atomistic model of the planar interface between Au-fcc and Ge(001) was also proposed.

First principles calculations of the cohesive energy and elastic constants as well as phonon dispersion relations show stability of both hcp and dhcp polytypes of gold [24]. Ab initio studies indicate higher stability of the fcc phase and a tendency towards hcp → fcc phase transformation. However, the calculated differences between the polytypes are very small [14,25]. In contrast, molecular dynamics simulations of metallic nanowires show a phase transformation from fcc to hcp below a critical diameter [26]. These findings support the feasibility of obtaining new hexagonal structures under conditions where surface effects are significant.

The main aim of our study was to analyse the atomic ordering in different planar Au/Ge interfaces, characterize their energetic properties and present the accompanying changes in the electronic structure. To this end, the concepts of interfacial energy and of the work of separation (adhesion) were used. We applied first principles methods based on density functional theory (DFT) to study Au/Ge heterostructures with different interfacial plane orientations.

The remainder of the paper is organized as follows. In the Methodology section the calculation details are given, and the investigated crystal planes and possible interfaces are introduced. Section Results and Discussion begins with convergence tests and calculations conducted for several low-index surfaces and simple Au/Ge interfaces. We then proceed to discussing several variants of a heterostructure with parallel Ge(001) and Au-fcc(011) planes. To the best of our knowledge, only one of the structures, with [110]Ge||
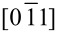
Au-fcc, has been observed experimentally [23]. We compare our results to the experimental data. We also investigate a novel hcp structure that is adjacent to the Ge(111) surface. From the electron microscopy image [23] one can identify the Au-hcp plane parallel to Ge substrate as (010). We discuss the optimized structures and defects that could stabilize the interface. Finally, we demonstrate the electronic properties of Au/Ge junctions and the formation of Ge–Au bonds at the interface. The Appendices provide surface energy details for gold and germanium crystals and the charge density differences for the Au/Ge heterostructures.

## Methodology

### Interfacial energy and the work of separation in DFT calculations

In contrast to the bulk phase of a material, the surface atoms have an incomplete set of neighbors and, therefore, unrealized bonding energy (surface energy). When two crystalline solids bind together, the atoms or molecules form an interface with preferred plane orientations determined by the strength of bonding, which can be quantified using the concepts of interfacial free energy and the work of separation. Their values can be obtained from DFT calculations of the total energies of appropriate systems modeling the heterostructure as well as the bulk crystals and slabs with a vacuum layer. The methodology details (from choosing the interface model up to the optimization methods) differ between implementations [12,27]. Here we focus on the approach in which two crystalline solids (*X* and *Y*) form a supercell with imposed periodic boundary conditions, that is, there are two interfaces within the supercell. The interfacial energy γ_int_ is defined as:


[1] $$\gamma_{\mathrm{int}}=\frac{1}{2A_{\mathrm{int}}}\left[E_{XY}-\left(n_{X}\varepsilon_{X}^{\text{bulk}}+n_{Y}\varepsilon_{Y}^{\text{bulk}}\right)\right],$$

where *E_XY_* is the total energy of the *XY* heterostructure, 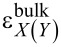 is the energy per atom in the *X*(*Y*) bulk form, *n_X(Y)_* is the number of *X*(*Y*) atoms in the *XY* supercell and *A*_int_ is the area of the interface.

The work of separation (*W*_sep_) is defined as the energy required to reversibly separate a bulk material into two semi-infinite bulks with free surfaces. Here we calculate this energy for an *XY* heterostructure, separated into two slabs, that is, two supercells with either *X* or *Y* atoms and a vacuum layer, with periodic boundary conditions imposed:


[2] $$W_{\text{sep}}=\sigma_{X}+\sigma_{Y}-\gamma_{\mathrm{int}},$$

where σ*_X_* and σ*_Y_* are the surface energies of *X* and *Y* phases. Their values can be obtained from the following formula:


[3]

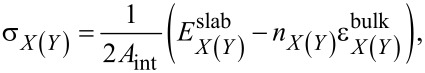



where 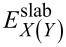 is the total energy of the *X*(*Y*) slab with two free surfaces.

All calculations are based on the DFT method implemented in the plane wave basis VASP code [28–29]. The Perdew–Burke–Ernzerhof (PBE) functional [30] within the generalized gradient approximation (GGA) for the exchange and correlation energy was used. The electronic wave functions were expanded as linear combinations of plane waves, truncated to include only plane waves with kinetic energies below a cutoff energy *E*_cut_ = 350 eV. The valence states were optimized with the Ge(*s*^2^*p*^2^) and Au(*s*^1^*d*^10^) electron configurations.

Bulk materials, surfaces and interfaces were simulated using the supercell method with periodic boundary conditions. Optimizations of the structural parameters (lattice constants and atomic positions) were carried out using the Monkhorst–Pack grid of **k**-points appropriate for the calculated structure: from a dense (8,8,8) grid for crystallographic cells of bulk crystal to a (4,4,4) grid for larger superlattices that model the Au-fcc(011)/Ge(001) and Au-hcp(010)/Ge(111) heterostructures. The slabs with vacuum were calculated using (8,8,2) or (4,4,2) **k**-points grids depending on the size of the cross section. A vacuum layer of 15 Å was selected for each structure to eliminate interactions between the two surfaces. The conditions for ending the optimization loops for electronic and ionic degrees of freedom were defined by the total energy difference between the steps of 10^−8^ eV and between the internal forces of 10^−2^ eV·Å^−1^, respectively.

In all calculations, the starting configurations (Au/Ge supercells, bulk structures and isolated slabs) were prepared under the assumption that the lateral extent of the gold fraction of the heterostructures matches the optimized dimensions of the germanium lattice, treated here as a substrate. The lattice constant perpendicular to the interface as well as the atomic positions are fully relaxed. The calculated interfacial energies capture mainly the chemical bonding energy at the interface and the energy cost of internal distortions since lattice mismatch strains occur both in the bulk and in the supercell configurations.

### Structural models

Under ambient pressure, germanium and gold crystallize in the diamond (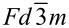) and the fcc (*Fm*3*m*) structure, respectively. Their experimental lattice constants are *a*_Ge_ = 5.66 Å for germanium and *a*_Au−fcc_ = 4.08 Å for gold [31]. The hexagonal hcp phase of Au, formed during specific processes of annealing and cooling of gold and germanium, is characterized by the *P*6_3_/*mmc* space group with lattice constants *a*_Au−hcp_ = 2.90 Å and *c*_Au−hcp_ = 4.88 Å [23]. These structural parameters are well reproduced in our DFT calculations. The obtained cubic lattice constants are 5.77 Å and 4.16 Å for Ge and Au-fcc, respectively, and the hexagonal lattice parameters are 2.93 Å and 4.89 Å for Au-hcp.

**Figure 1:**
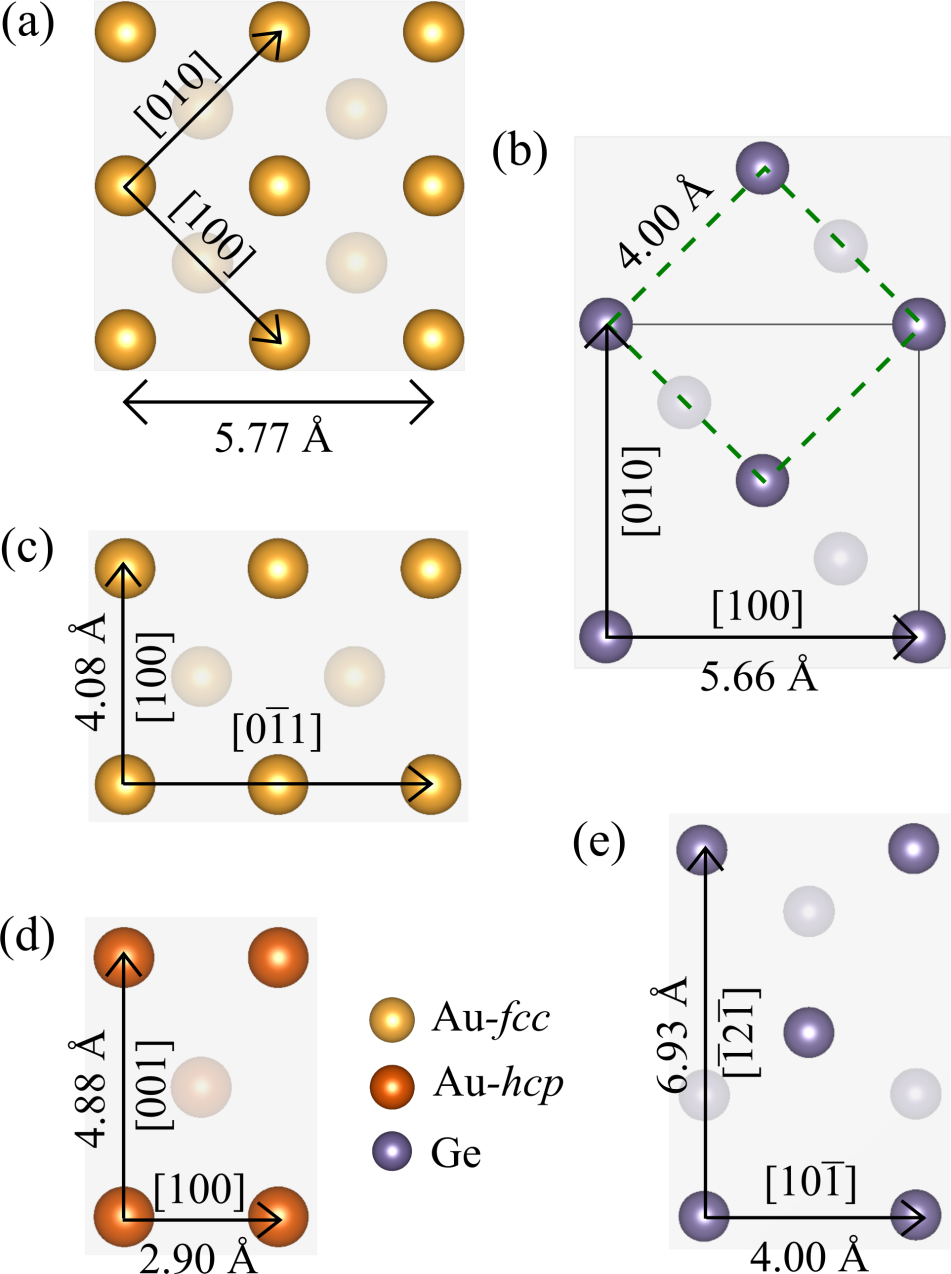
Surfaces of Au and Ge slabs investigated in our study, with experimental interatomic distances: (a) Au-fcc(001); (b) Ge(001), dashed green square indicates the building blocks matching the [100]Au length; (c) Au-fcc(011); (d) Au-hcp(010), or (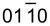) in the Miller–Bravais notation; (e) Ge(111). To distinguish the hcp lattice, the Au atoms are shown in dark orange.

Figure 1 shows surfaces that terminate the slabs that are the building blocks for heterostructures investigated in this study. We constructed them by looking for matching lattice parameters and following the experimental data if available. Moreover, different mutual positions of the slabs were considered in order to find the best atomic arrangement.

It can be easily noticed that (001) faces of Au-fcc and Ge can form an almost strain-free heterostructure if the gold slab as in Figure 1a is joined with the Ge slab marked with a black square in Figure 1b. A variant of such a superlattice was used to perform convergence tests described in the next section.

Another way of exploiting this lattice match is to connect a Au-fcc(011) plane as shown in Figure 1c and the same Ge(001) slab. We note that now we get a strain-free interface only in one direction, and we need a few Au and Ge slabs to reduce the lattice strain in the perpendicular direction. Alternatively, the Au-fcc(011)/Ge(001) heterostructure can be built using a Ge slab marked with the green square, as its side length matches the gold lattice constant. Again, multiple cells are needed in the perpendicular direction.

There is no obvious way to build the Au-hcp/Ge interface using low-index Au-hcp and Ge surfaces. Thus, we decided to make calculations for the experimentally found Au-hcp(010)/Ge(111) interface only. To model the heterostructure, we multiply the relevant Au and Ge surfaces (shown in Figure 1d,e) to obtain minimal strain within a reasonable size of the system.

## Results and Discussion

### Convergence tests

**Figure 2:**
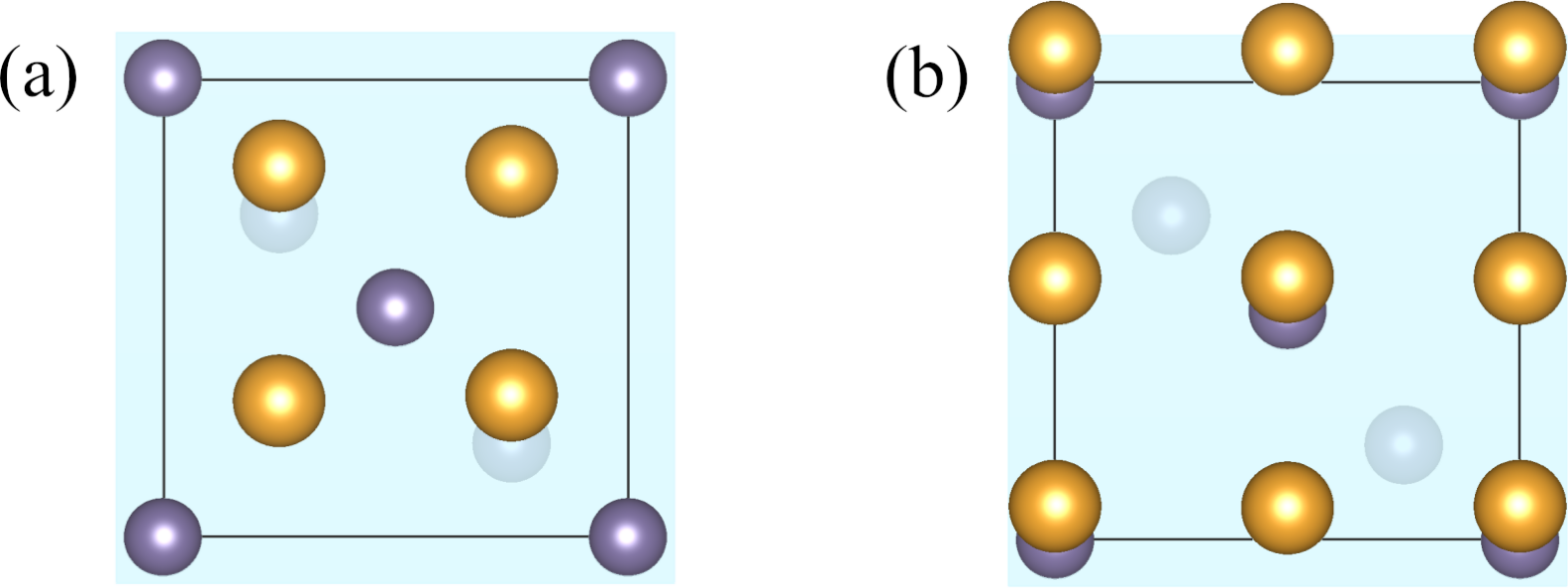
Two variants of Au-fcc(001)/Ge(001) interface atomic arrangement: (a) variant *T*_1_ with the interfacial Au atoms above bridge sites of Ge surface (this structure was used for convergence tests) and (b) variant *T*_2_ with Au atoms directly above the surface Ge atoms. The shaded surface indicates the interface Ge layer, and the second Ge layer is visible below (crystal planes are slightly tilted to show all the atoms).

Ideally, the thickness of the slabs should be large enough to ensure that all the surface effects are captured within the supercell and that the two interfaces resulting from periodic boundary conditions do not interact with each other. To test the convergence of surface energies, interface energies and the work of separation, we considered Ge(001) and Au-fcc(001) slabs with different numbers of atomic layers and the epitaxial Au-fcc(001)/Ge(001) heterojunction shown in Figure 2 (two variants of mutual positions of the slabs are presented). This simplest heterostructure can be built by setting the [110] direction of the Au crystal parallel to the [100] direction of the Ge lattice [32], that is, by combining two fragments of the lattice planes oriented as in Figure 1a,b. The resulting mismatch ε defined as


[4] $$\varepsilon =\left(a_{\text{Au}-fcc}\sqrt{2}-a_{\text{Ge}}\right)/a_{\text{Ge}}\times 100\%,$$

is only 2.0%, that is, the heterostructure is almost strain-free. As only four Au atoms and two Ge atoms form a single layer of this interface, one can easily increase the number of layers in the supercell. In variant *T*_1_ of the considered heterostructure, the Au atoms are at maximum distance from the Ge atoms, while in variant *T*_2_ some of the gold atoms are located on the top of Ge sites.

**Figure 3:**
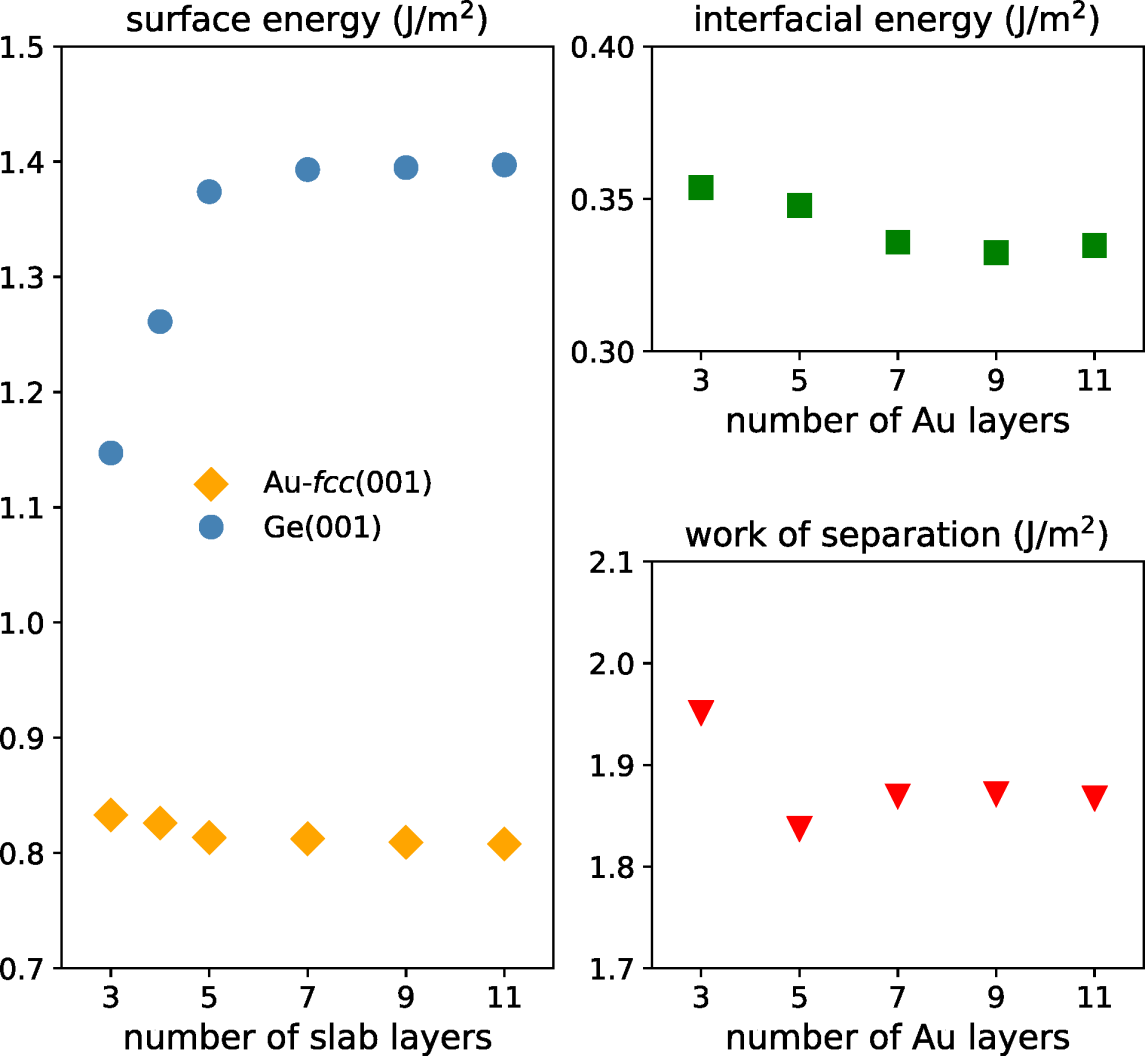
Convergence tests: (left) dependence of Au-fcc(001) and Ge(001) surface energies on the number of slab layers, (right) the interfacial energy and work of separation for the Au-fcc(001)/Ge(001) heterojunction vs the number of Au layers (the number of Ge layers is five).

Our preliminary calculations indicated that variant *T*_1_ is preferred, and we conducted convergence tests for this structure. First, separated slabs of Ge and Au-fcc crystals terminated with (001) planes and a vacuum layer of 15 Å were used to determine the optimal slab thicknesses for surface energy calculations. The calculated values plotted in Figure 3 (left panel) show that above the fifth layer the discrepancies between results are smaller than 0.03 J/m^2^. The large initial increase of the surface energy values seen in the data for Ge crystal results from the very small number of atoms (only six) in this slab and the two free surfaces.

The right panels of Figure 3 present the convergence tests performed to find the optimal thickness of the Au layer in the Au/Ge heterostructure with five Ge layers. The plot of the interfacial energy reveals a much weaker dependence on the number of Au layers. Values of the work of separation for the Au-fcc(001)/Ge(001) heterojunction include both the calculated surface and interface energies. Hence, we observe again differences in the results of about 0.03 J/m^2^ for slabs with more than five layers of Au. We conclude that five layers of both Au and Ge atoms in the slabs are sufficient for our calculations. This observation is important for the investigation of more complicated interfaces where larger numbers of layers result in very long computational times.

### Results for model surfaces and interfaces

**Figure 4:**
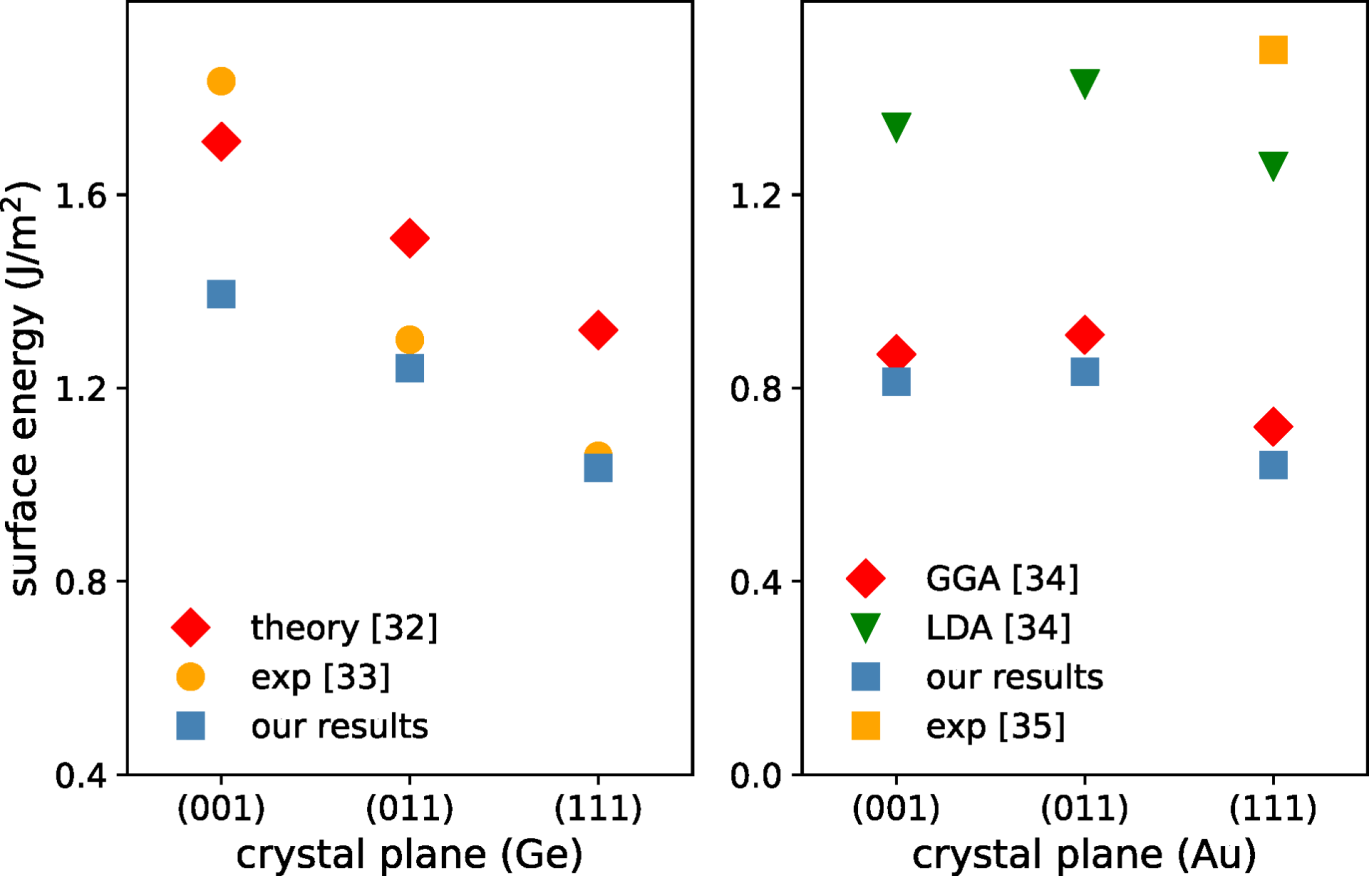
Surface energies calculated for germanium (left) and Au-fcc right) crystal planes compared to available experimental and theory data (values are given in Appendix A).

We consider several low-index planes of Ge, Au-fcc and Au-hcp crystals (some of them are presented in Figure 1). The calculated surface energies are presented in Figure 4 and Appendix A. The comparison of our results with the available theoretical and experimental data shows that different theoretical approaches lead to discrepancies in absolute values of surface energies; however, the relative changes between the investigated crystal planes are very similar. Experimental data for Ge crystals show the same trend, that is, a decrease of the surface energy from (001) to (111) planes, with a slightly bigger slope.

**Figure 5:**
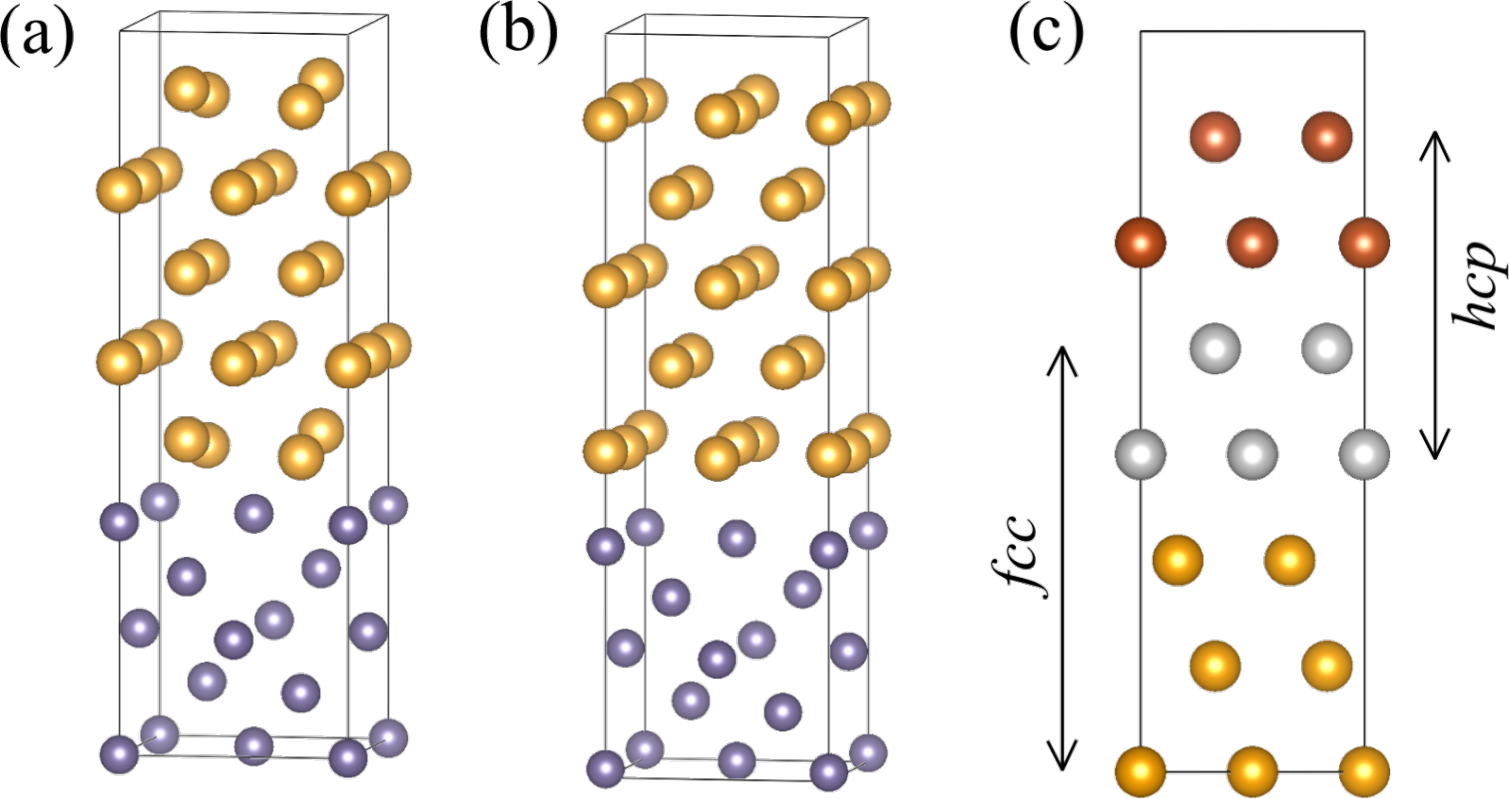
The relaxed structures of the simplest coherent interfaces. Two variants of the Au-fcc(001)/Ge(001) heterostructure: (a) variant *T*_1_ with interfacial Au atoms at bridge sites of Ge surface and (b) variant *T*_2_ with interfacial Au atoms at top sites of Ge surface. Panel (c) shows the Au-fcc(001)/Au-hcp(0001) interface with the two phases of gold marked in yellow and dark orange. The positions of the gray atoms are compatible with both arrangements.

The optimized atomic arrangements for our model Au-fcc(001)/Ge(001) heterostructures are shown in Figure 5 (variants *T*_1_ and *T*_2_). The total energies of the two considered supercells are −108.806 eV and −106.484 eV for the variants *T*_1_ and *T*_2_, respectively. Therefore, the realization of variant *T*_1_ with interfacial Au atoms located at bridge sites of the germanium surface is more probable. In Table 1 we present the calculated values of interface energy and work of separation together with the structural parameters useful for describing interfaces. These parameters are (i) the intralayer rumpling parameter (*r*_Ge_ and *r*_Au_), defined as the maximal difference between the *z*-th coordinates of atoms belonging to one layer, and (ii) the interlayer distance (*d*_int_). Comparing variants *T*_1_ and *T*_2_, we notice the big discrepancy between their interfacial energies (−0.6 J/m^2^). The favored *T*_1_ structure is characterized by a larger Au rumpling parameter, arising from interactions between interfacial Au atoms with atoms from the next Ge layer, and a smaller interlayer distance compared to variant *T*_2_.

**Table 1:** Interfacial energies (γ_int_), work of separation values (*W*_sep_), rumpling parameters *r*_Ge_ (*r*_Au_) for interfacial Ge (Au) layers and interlayer distances at the interface (*d*_int_) calculated for different Au/Ge heterostructures as well as for the Au-fcc/Au-hcp superlattice.

interface	variant	γ_int_ (J/m^2^)	*W*_sep_ (J/m^2^)	*r*_Ge_ (Å)	*r*_Au_ (Å)	*d*_int_ (Å)

Au-fcc(001)/Ge(001)	*T*_1_	0.345	1.862	0.00	0.40	1.76
	*T*_2_	0.909	1.298	0.00	0.13	2.36
Au-fcc(111)/Au-hcp(001)	–	0.041	1.411	–	0.00	2.41
Au-fcc(011)/Ge(001)	*A*	0.590	1.638	0.08	0.53	1.55
	*B*	0.821	1.407	0.49	0.38	1.93
	*C*	0.336	1.892	0.21	0.61	1.77
	*D*	0.437	1.784	0.29	0.62	2.13
Au-hcp(010)/Ge(111)	–	0.369	1.506	0.23	0.30	2.17

The Ge–Au bond lengths obtained for the *T*_1_ structure, 2.50 Å, are in good agreement with the experimental values for amorphous GeAu alloys, 2.66 Å, [33] and thin films of Au covering a Ge(111) surface, 2.5 Å, [34]. In variant *T*_2_ the Au–Ge distance between Au atoms lying directly above Ge atoms, *d*_int_ = 2.36 Å, is shorter than Ge–Au bond lengths reported previously. The preferential localization of Au atoms at bridge sites rather than at top sites of Ge surfaces should be taken into account when modeling other interface models.

We conclude the discussion of model heterostructures with results for the fcc(111)/hcp(001) interface with perfect match, shown in Figure 5c. It changes the ABC fcc arrangement of lattice planes into the ABAB pattern, and, as can be seen in the figure, one cannot uniquely assign the two planes to either of these phases. We symmetrically divided the gray layers between the two phases and calculated the interface energy values. As could be expected, the value of the Au-fcc/Au-hcp interface energy is much smaller (by an order of magnitude) than those of the other interfaces. The work of separation includes also the surface energy and is of the same order of magnitude as in other considered interfaces.

### Experimentally observed heterostructures

In this section we present our results of ab initio calculations for the Au/Ge interfaces observed in experimentally grown gold nanoislands on a germanium substrate [23]. Available STEM-HAADF images demonstrate the presence of well-defined strain-free planar interfaces formed between gold and germanium substrate, in which epitaxial relationships can be identified. A particularly regular Au-fcc(001) structure can be found on the Ge(001) surface. It is characterized by a parallel arrangement of the Au[100] and the Ge[110] directions, with a near perfect match between the lattice distances. When the sample is annealed above the eutectic temperature, a Au-hcp(010) phase facing the Ge(111) plane is also observed. These observations indicate that the interface bonding between Au and Ge phases can be modelled by the supercell geometry.

#### Au-fcc(011)/Ge(001)

**Figure 6:**
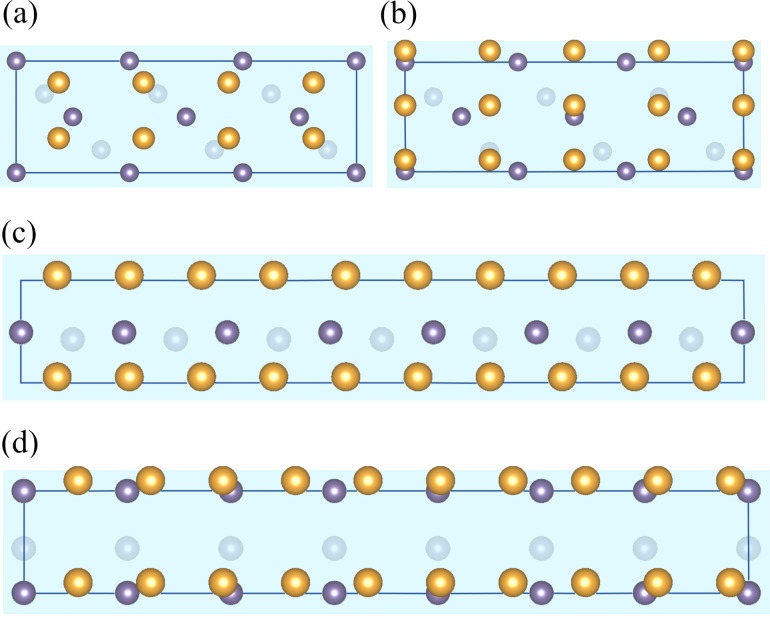
Variants *A*, *B*, *C* and *D* of the Au-fcc(011)/Ge(001) interface (initial configurations) are shown in panels (a–d), respectively. (a, b) Four Au slabs as shown in Figure 1c are placed upon a Ge supercell constructed from three blocks marked with a black square in Figure 1b. In variant *A* the interface Au atoms avoid Ge atoms, while in variant *B* some of the Au atoms are placed directly above Ge atoms. (c, d) Five Au slabs as shown in Figure 1c are joined with seven Ge blocks marked by a green square in Figure 1b. Variants *C* and *D* of this interface differ in orientation of the Ge slabs with respect to the Au slabs.

As already discussed, there are several possibilities of building a low-energy Au-fcc(011)/Ge(001) heterostructure, and here we compare their calculated interface energy values and structural parameters. First, we join the Au-fcc(011) plane oriented as in Figure 1a and the Ge(001) plane from Figure 1b, using a cell marked with the black square as a building block for the supercell. In such a way, we can get an almost strain-free heterostructure in the Au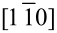 direction. In order to reduce strain along the perpendicular Au[100] direction, we take four Au lattice constants and three Ge blocks (the resulting mismatch is about 4%). To get benchmark results for this orientation of crystal planes, we consider two different mutual positions of the slabs presented in Figure 6a and Figure 6b.

In variant *A*, rows of gold atoms are placed between the rows of germanium atoms, and we can expect from previous calculations that this arrangement is energetically preferable to interfaces in which some Au and Ge atoms are forced to make shorter bonds. We include five Au and five Ge layers in the supercell, which gives altogether 70 atoms (30 Ge and 40 Au). Similarly to the Au(001)/Ge(001) interface, the gold atoms close to the interface move slightly from their initial positions (rumpling parameter *r*_Au_ = 0.53 Å), while the stiffer germanium lattice stays almost unchanged (rumpling parameter *r*_Ge_ = 0.08 Å). In variant *B*, a shift of the Au plane with respect to the Ge surface that places the column of Au atoms right over the Ge atoms (Figure 6b) leads to a significant increase in the total energy of the system (supercell) by about 3.5 eV. Additionally, the forces induced to preserve the Ge–Au bond length on the appropriate level lead to an increase of the rumpling parameter of the Ge layer up to 0.49 Å. The interface energy of variant *B* is higher than that of variant *A* by 0.23 J/m^2^. These findings confirm that structures with some Au atoms located directly above the Ge atoms tend to be energetically expensive.

Atomically resolved STEM-HAADF images of gold nanoislands on germanium substrates [23] suggest yet another way of matching Au-fcc(011) and Ge(001) crystal planes. Since the distance between germanium atoms in the [110] direction closely matches the gold lattice constant (mismatch of about 1.9%), we can use the green square in Figure 1b as a new building block for the heterostructure.

We note that there are two different orientations of this block with respect to the Au-fcc(011) slab shown in Figure 1c. Both arrangements are consistent with electron microscopy pictures as long as the Au and Ge atoms at the interface are not discriminated. In the perpendicular direction (with no experimental image to guide us), we chose to join seven germanium blocks and five gold slabs as shown in Figure 1c, obtaining a mismatch of about 3%. Variant *C* is constructed in such a way that rows of gold atoms are located between the germanium rows (see Figure 6c), and we expect this arrangement to be energetically favored. Indeed, the interface energy obtained for a supercell containing six layers of Ge (42 atoms) and five layers of Au (50 atoms) is equal to 0.336 J/m^2^, a value very close to the lowest energy obtained earlier for the optimal Au-fcc(001)/Ge(001) structure.

**Figure 7:**
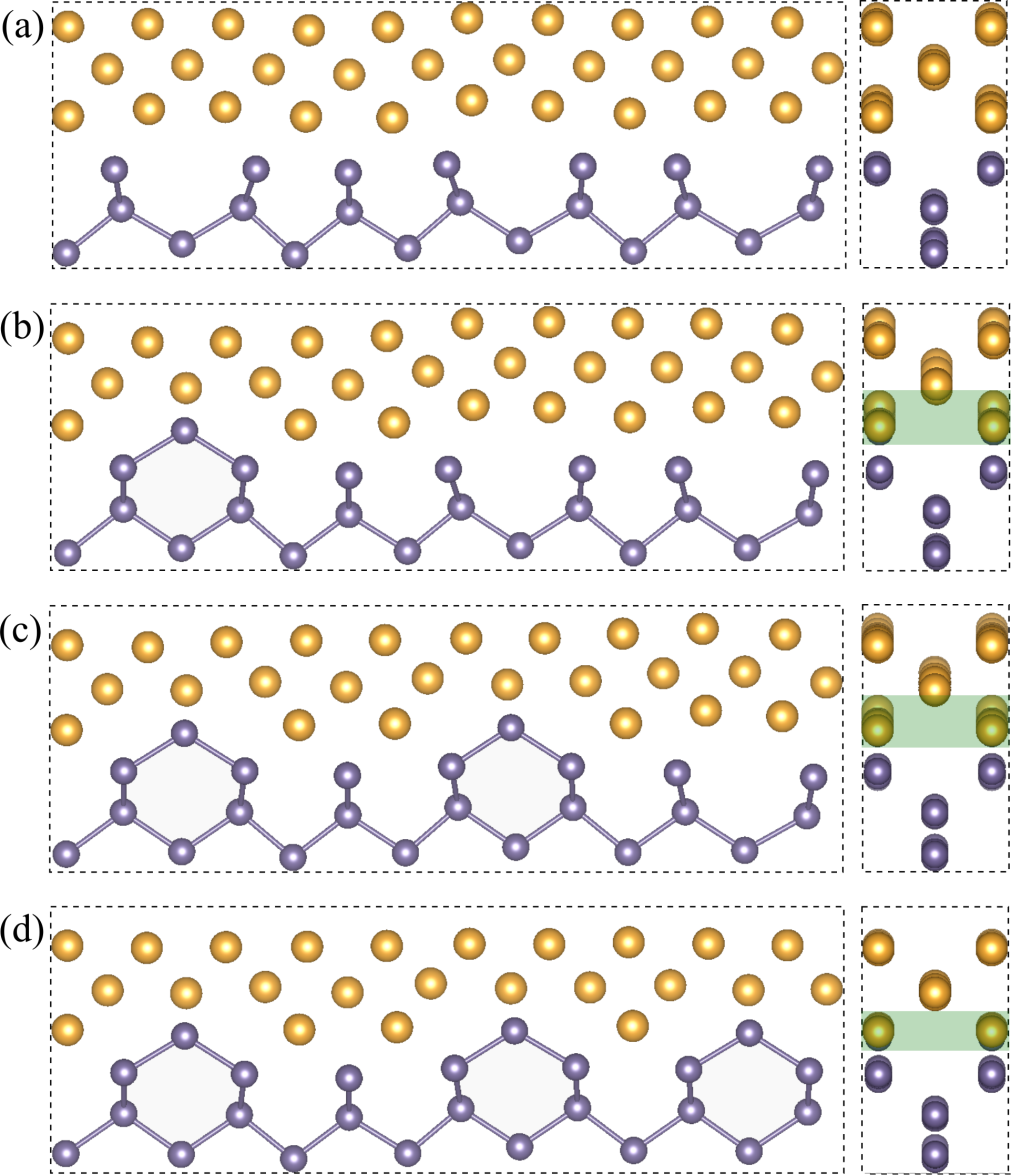
Introduction of defects into variant *D* of the Au-fcc(011)/Ge(001) heterostructure: (a) optimized structure of variant *D*; (b–d) additional Ge atoms per supercell introduced to the gold layer from which some Au atoms are removed (twice as many as the number of Ge atoms added). The green rectangle marks the extension of the mixed Au/Ge layer. For better visibility of the changes in the structure, Ge–Ge bonds are shown in the left panels.

Small displacements of atoms from initial positions are observed in the interface layer (*r*_Au_ = 0.62 and *r*_Ge_ = 0.22 Å), and the distance between Ge and Au interfacial planes is equal to 1.78 Å (again, very similar to our first low-energy heterostructure). These numbers suggest that variant *C* is a good candidate for the observed nanostructure. However, a detailed analysis of the scattered intensity of atomic columns [23] suggests that the rows of Au atoms are located directly above the rows of Ge atoms. We therefore built variant *D* of the heterostructure, with 102 atoms (42 Ge and 60 Au), and the interface shown in Figure 6d. In order to match the experimental picture, we need a different orientation of the germanium building blocks (rotation by 90° with respect to variant *C*). Due to incommensurate Au–Au and Ge–Ge distances, it is impossible to find a regular pattern of Au–Ge bonds in the resulting interface layers (some of them might be much shorter than in the optimal Au/Ge arrangement). After optimization, we get similar values of rumpling parameters as in variant *C*, but the distance between Au and Ge layers is increased by almost 0.4 Å. Because of the different numbers of atoms in each supercell we cannot make a direct comparison of the total energies; however, the interface energy in variant *D* is higher by approximately 0.1 J/m^2^. It is still the second lowest value among the variants of the Au-fcc(011)/Ge(001) interface, and the difference with respect to the optimized variant *C* is relatively small. The slightly higher interface energy in variant *D* might arise from the distortions present in the Ge substrate visible in Figure 7a and the enlarged distance between the two phases.

**Figure 8:**
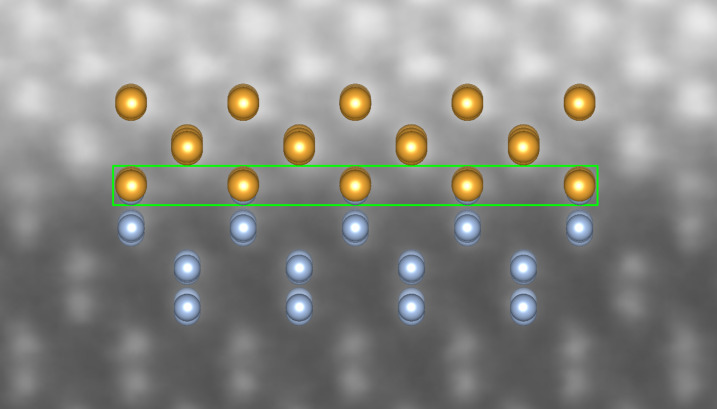
Experimentally observed Au-fcc(011)/Ge(001) interface: atomically resolved STEM-HAADF image [23] and atomic structure obtained for variant *D* with defects, view as in the right panel of Figure 7d, duplicated four times. The green rectangle marks the mixed layer.

In the last part of this section, we discuss the possibility of lowering the interfacial energy of this variant by introducing defects into the interface layer. We investigated three supercells of variant *D*, in which some pairs of Au atoms in the interface layer were replaced by Ge atoms. Their optimized atomic arrangements are presented in Figure 7b–d. We can observe that while one such defect, shown in Figure 7b, seems to disrupt the arrangement of some atoms even further, more defects restore a remarkably regular structure with a decreased distance between layers at the interface. The calculated interface energy values for the structures in Figure 7a–d are 0.437, 0.416, 0.388, and 0.343 J/m^2^, respectively. The Ge atoms replacing some Au pairs gradually improve the interface energy, and the result obtained for the last modification is very close to the interface energy of variant *C*. Figure 8 shows the experimental image of the investigated interface together with one of our optimized structures, shown in Figure 7d. We note, however, that variant *C* as well as variant *D* without defects would also be consistent with the available experimental image (showing only the view as in the right panels of Figure 7). Hence, we cannot use it to discriminate these arrangements.

#### Au-hcp(010)/Ge(111)

The last heterostructure considered here, including the hcp phase of gold, is the most interesting one from the perspective of investigating new phases in nanoscale structures and their possible applications. It is, however, also the most challenging interface for our ab initio calculations. The mutual position of the slabs can be partially read from electron microscopy images [23], and the identified gold lattice plane adjacent to the Ge(111) surface is (010), with Ge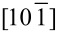 parallel to the Au-hcp[100] direction. An interface with relatively low strain can be formed with 4 × 3 Au-hcp(010) blocks connected to 3 × 2 Ge(111) blocks as shown in Figure 1 (the supercell contains 96 Au and 72 Ge atoms). The Au lattice is extended in the Au-hcp[100] direction (with a mismatch of about 3%) and compressed in the Au-hcp[001] direction (mismatch of 6%).

**Figure 9:**
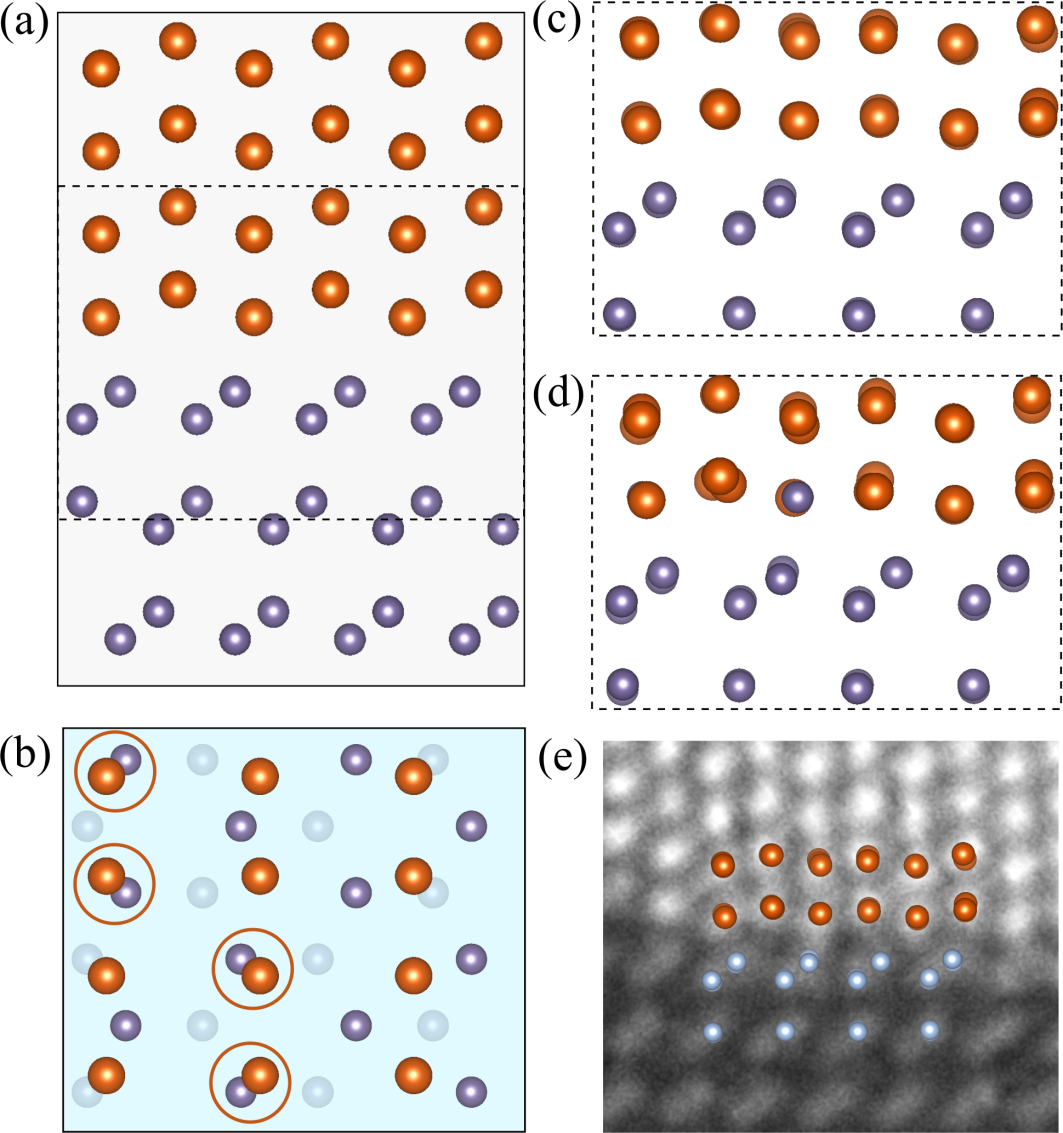
Initial configuration of the Au-hcp(010)/Ge(111) supercell: (a) side view and (b) top view of the interface. The relative position of Au and Ge slabs is selected so as to obtain the maximal possible distance between the nearest Au and Ge atoms. There are four such short distances per interface (i.e., eight in the supercell) indicated by circles. (c) Relaxed atomic pattern within the square in panel (a). (d) Relaxed structure in which Au atoms with the shortest Au–Ge distance are replaced by Ge atoms. (e) Atomically resolved STEM-HAADF image of the Au-hcp/Ge(111) interface [23] with the optimized supercell as shown in panel (c).

In this particular Au/Ge heterojunction, it is not possible to find a starting configuration with similar Au–Ge distances across the interface, and inevitably some of them are significantly shorter compared to other optimized structures. In the interface used for optimization, presented in Figure 9a,b, the mutual position of the slabs is chosen in such a way that the shortest Au–Ge bonds are maximal for a given distance between slabs, that is, extremely short bonds are avoided. It occurs that at each interface there are four short Au–Ge bonds, marked with circles in Figure 9b. As a consequence, DFT calculations with full atomic displacements lead to strong changes in the Au atomic positions. In the optimized structure shown in Figure 9c the characteristic hcp zigzags are flattened. The lattice strain present in the Au part of the heterostructure may also be a factor contributing to atomic displacements away from the hcp lattice positions.

The obtained value of interface energy (0.369 J/m^2^) is similar to the previously investigated variants *C* and *D* of the Au-fcc(011)/Ge(001) heterostructure. The work of separation (1.506 J/m^2^) is smaller because of the surface energies of the respective building blocks.

We considered also possible defects in the lattice that could lower the energy cost of the short Au–Ge bonds and better preserve the hcp ordering in the Au part of the slab. In one of the modifications, we introduced Ge atoms in place of Au atoms in the four positions at the interface where the Au–Ge distances are minimal. While the hcp zigzags are more pronounced in the optimized supercell shown in Figure 9d, the interface energy is slightly higher (0.395 J/m^2^) than that of the defect-free junction. We also tested another type of defects (Au vacancies). To construct the supercell, we first found the position of slabs with the smallest possible Au–Ge distances (two such pairs per interface can be found) and removed the Au atoms from these bonds. The optimized lattice preserves the hexagonal arrangement; however, the interface energy is significantly higher than in the other considered hcp variants (0.514 J/m^2^).

Figure 9e shows the experimental picture and the defect-free optimized structure shown in Figure 9a. We can observe that the atomic pattern at the interface is well reproduced by our model. The interface seen in the experimental image is not as regular as the Au-fcc/Ge junction presented in Figure 8, and other types of defects may additionally stabilize this heterostructure.

### Electronic structure

**Figure 10:**
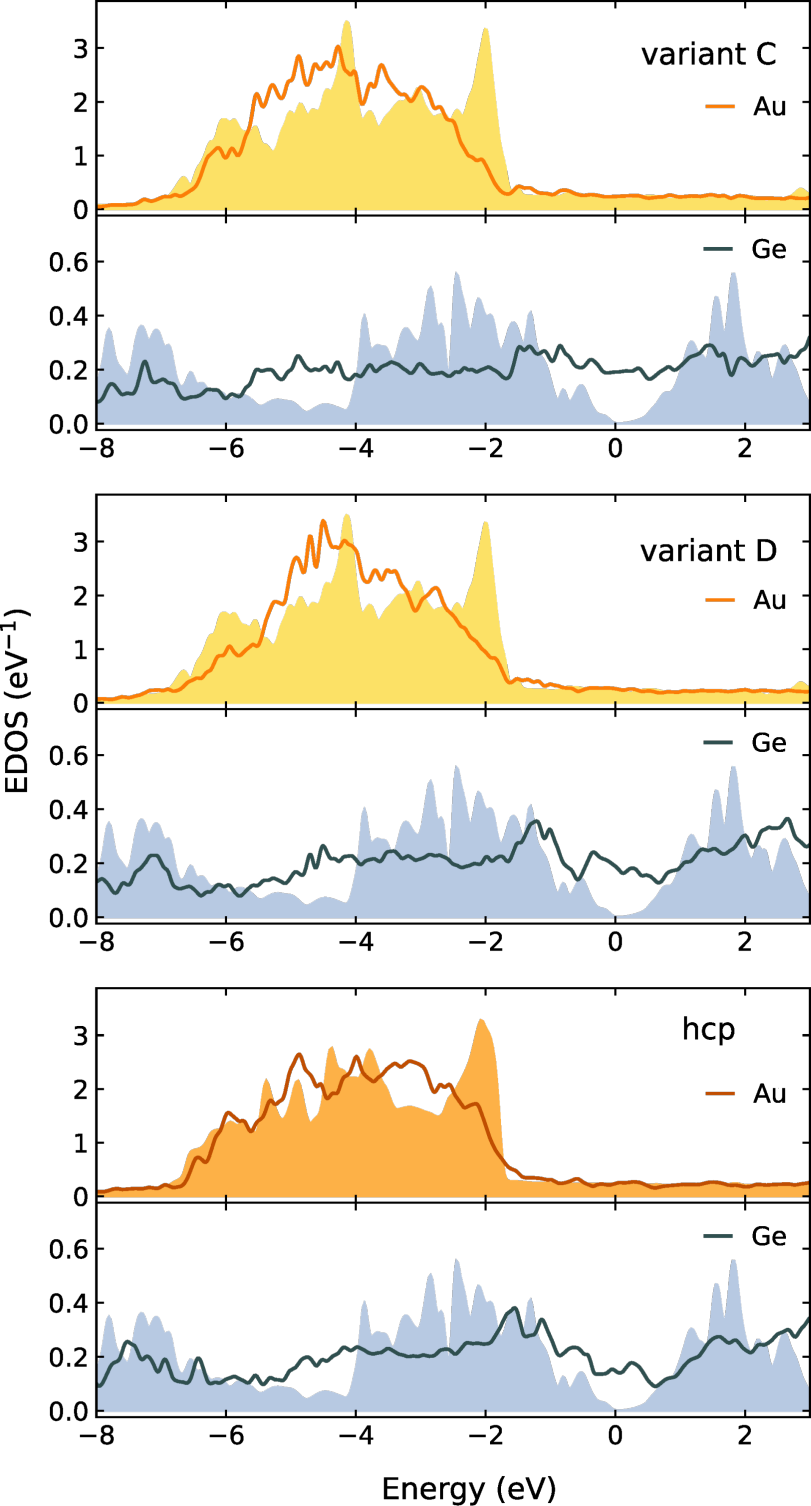
The electron density of states (EDOS) for Au and Ge atoms averaged over all atoms in layers adjacent to the interface. The results for the respective bulk phases are presented as filled areas. For all plots *E*_F_ = 0.

Here we discuss electronic properties of the optimized defect-free Au/Ge interface structures investigated in the previous section, that is, the variants *C* and *D* of the Au-fcc(011)/Ge(001) interface and the Au-hcp(010)/Ge(111) heterostructure shown in Figure 9a. Figure 10 presents the electron density of states (EDOS) for Au and Ge atoms (averaged over all atoms in layers adjacent to the interface). These results are compared to the bulk density of states. Small differences in the bulk phase between Au-fcc and Au-hcp can be seen from the plots. First of all, the electron density at the Fermi level is finite for the Ge atoms, making the interface region metallic (we observe this effect also for other Ge layers with only a slight decrease in the density of states with the distance from the interface). In contrast, the EDOS for gold atoms remains essentially unchanged around the Fermi level with respect to the bulk phase for all investigated interfaces. However, the big peak near −2 eV disappears, and the density is shifted towards lower energy values. This shift can be understood as formation of bonding states with germanium electrons.

**Figure 11:**
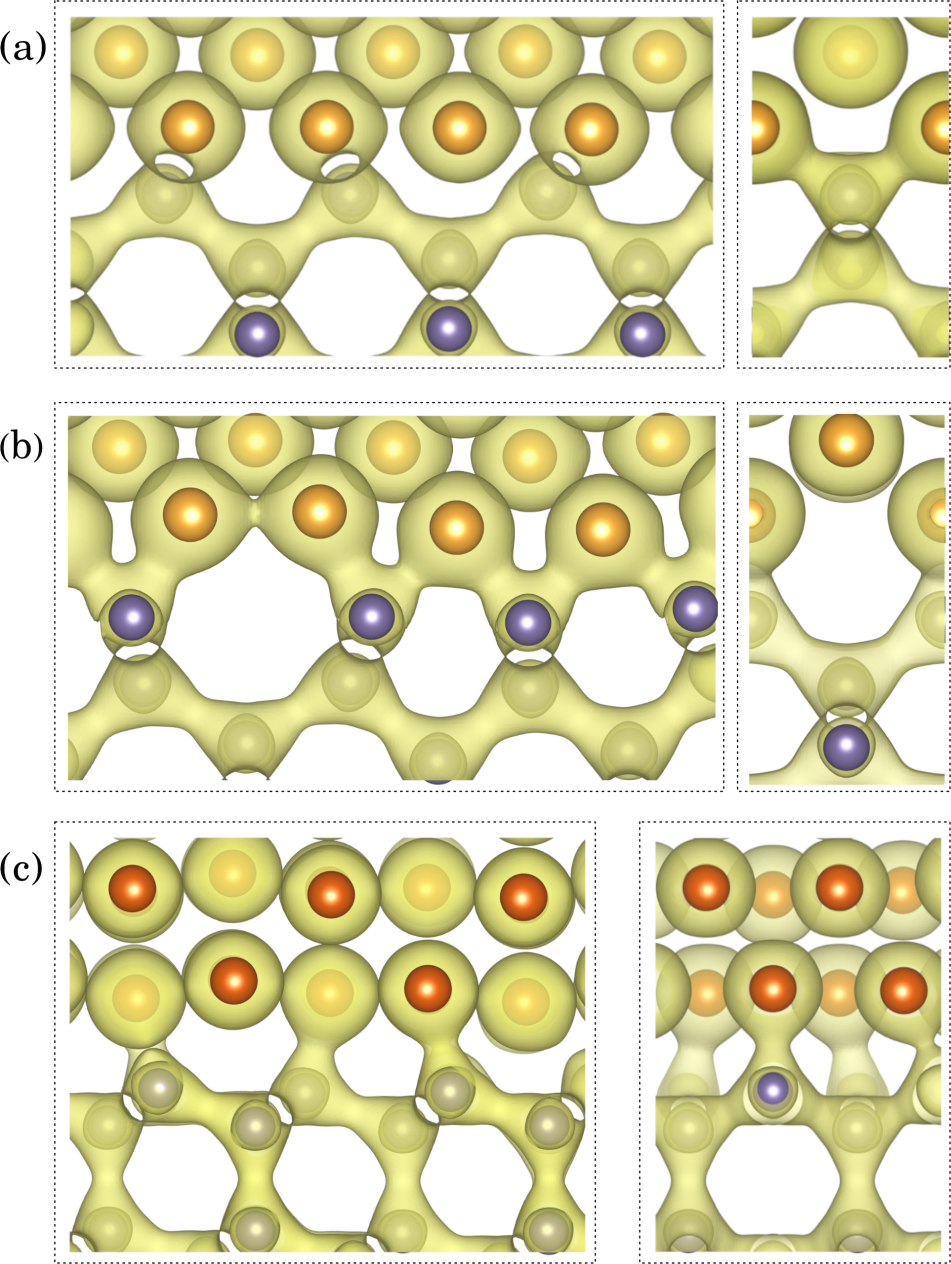
Isosurfaces of charge density (0.051 *e*/Å^−3^) for variants (a) *C* and (b) *D* of the Au-fcc(011)/Ge(001) interface, compared with the isosurface of the Au-hcp(010)/Ge(111) interface (c).

Emergence of Ge–Au bonding in the modeled heterostructures can be illustrated by plots of charge distribution at the Au/Ge interface. Figure 11 presents isocharge surfaces (0.051 *e*/Å^−3^), connecting pairs of atoms if a bond is formed between them. Away from the interface, each germanium atom shares four covalent bonds with its nearest neighbors, and gold atoms form a metallic phase. At the interfaces, bonding states can also be seen between Ge and Au atoms. In the Au-fcc(011)/Ge(001) interfaces each Ge atom is attached to two Au atoms, while the Ge atoms at the Au-hcp(010)/Ge(111) interface are bonded with one Au atom (the difference arises from different number of unbound germanium electrons in a given crystal surface).

**Figure 12:**
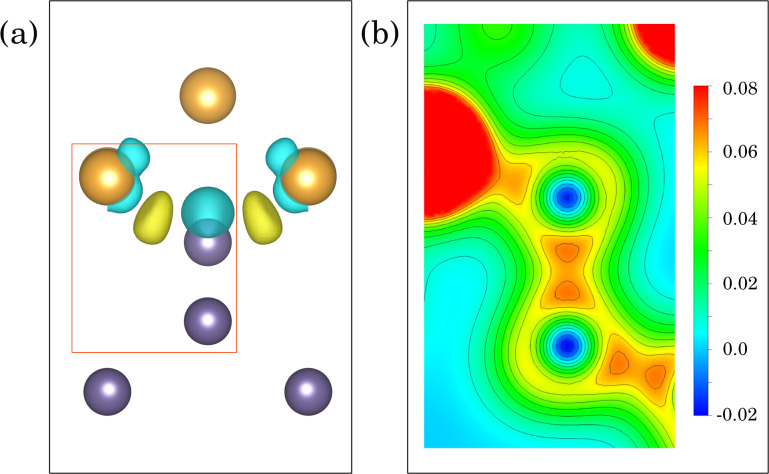
(a) Isosurfaces of the charge density difference for variant *C* at 0.0064 *e*/Å^−3^ (yellow and blue correspond to positive and negative values, respectively). (b) The map of charge density distribution for a plane defined by the three atoms in the red frame of panel (a). The orientation of this plane is different from the view in panel (a).

Even deeper insight into the formation of these bonding states can be gained by investigating the charge density and charge redistribution at the interface. Figure 12a shows the charge accumulation (yellow) and charge depletion (light blue) regions with respect to separate Ge and Au slabs terminated with vacuum (see Appendix B for details). One can observe charge accumulation between the interface Ge and Au atoms, as expected for bond formation. Figure 12b presents the charge density map for a plane defined by three atoms inside the red frame in panel Figure 12a, that is, a cross section through both Au–Ge and Ge–Ge bonds, revealing their detailed structure. Perhaps unexpectedly, the charge density between the interface Au and Ge atoms reaches almost the same level as for Ge–Ge covalent bonds, see Figure 12b. The integrated charge density of the bonding electrons, however, can be expected to be significantly smaller. Also, one can observe that the Au–Ge bond is strongly asymmetrical. Similar bonding states were already observed for Au atoms deposited on a Ge(001) surface using X-ray photoelectron spectra, supported by first principles calculations [35].

## Conclusion

We have used density functional theory to study the structure and stability of phase boundaries found in gold nanoislands grown on Ge substrates. Because of the presence of epitaxial relationships between Au-fcc and Ge(001) or Au-hcp and Ge(111), confirmed by atomically resolved experimental images, we model the Au-fcc/Ge and Au-hcp/Ge junctions as supercells with planar interfaces. While we are not able to include all strains and deformations present in the nanostructures, our results capture the physics of interfaces in these materials and, thus, contribute to understanding their properties. We focused on the interfacial energy values that in our approach mostly capture the bonding energy of the interface atoms and can be used to predict the possibility of interface formation. We believe that the presented approach provides helpful insight into the configuration of the interface and energy cost of point defects present in studied heterostructures.

The calculations for the Au-fcc(011)/Ge(001) interface reveal the lowest interfacial energies for variants *C* and *D* of heterojunctions, consistent with available microscopic images. Refined experimental data indicating that variant *D* (with slightly higher interface energy) is realized led us to search for possible defects in the interface layer that might lower the energy cost of this junction. The optimized supercell is characterized by both a low interfacial energy and a regular atomic pattern. Our results suggest that the Ge(001) substrate induces the growth of the Au-fcc phase.

We also constructed several supercells for studying the novel hcp phase of gold. The interface energy of the optimized structure is close to the values obtained for variants *C* and *D* of Au-fcc/Ge interfaces. However, significant atomic displacements were found that result mainly from an uneven distribution of Au–Ge distances and can be reduced if the point defects are included. The poor matching of Au-hcp and Ge(111) structures indicates that other defects (dislocations or steps) should be also considered.

Electron density of states calculations and charge density distribution maps performed for the experimentally observed heterostructures demonstrate that the interface region becomes metallic; yet, covalent-like Ge–Au bonding states between the interface atoms are also formed. Each interfacial Ge atom forms two bonds with Au atoms in the Au-fcc/Ge structure and only one at the Au-hcp/Ge interface. The maps of charge density differences show charge transfer occurs both at the interface and in the adjacent germanium layer.

## Appendix A

### Surface energies

The surface energies calculated for several planes of Ge and Au-fcc crystals are given and compared to available theoretical and experimental data in Table 2. We also present results for the hcp phase of gold, considering the hexagonal (001) plane as well as the (010) plane relevant for our experimental interface models. It is worth noticing that the differences between the Au-fcc(111) surface and the Au-hcp(001) surface begin from the third layer only. This explains why the numerical results are almost identical for these planes. Generally, a surface with lower energy is usually considered more stable since it needs a smaller amount of work to be done when cleaving along this surface. Thus, the Ge(111) and Au-fcc(111) free surfaces are favored.

**Table 2:** Surface energies calculated for various low-index planes of Ge, Au-fcc and Au-hcp crystals. For comparison previous theoretical results and experimentally derived values are presented.

	surface energy (J/m^2^)		

Ge diamond	(001)	(011)	(111)
this work	1.394	1.241	1.035
theory (LDA) [38]	1.710	1.510	1.320
exp [39]	1.835	1.300	1.060
Au-fcc	(001)	(011)	(111)
this work	0.813	0.834	0.641
theory (GGA) [40]	0.87	0.91	0.72
theory (LDA) [40]	1.34	1.43	1.26
exp [41]	—	—	1.5
Au-hcp	(001)	(010)	
this work	0.639	0.840	

## Appendix B

### Charge density differences

The changes in charge distribution induced by the Au/Ge interface can be demonstrated by calculating the difference between the total charge density of the interface (ρ_Au/Ge_) and the charge densities of systems with vacuum substituting for Au (ρ_vac/Ge_) or Ge (ρ_Au/vac_) according to the formula:


[5] $$\Delta \rho =\rho_{\text{Au/Ge}}-\rho_{\text{vac/Ge}}-\rho_{\text{Au/vac}}.$$

**Figure 13:**
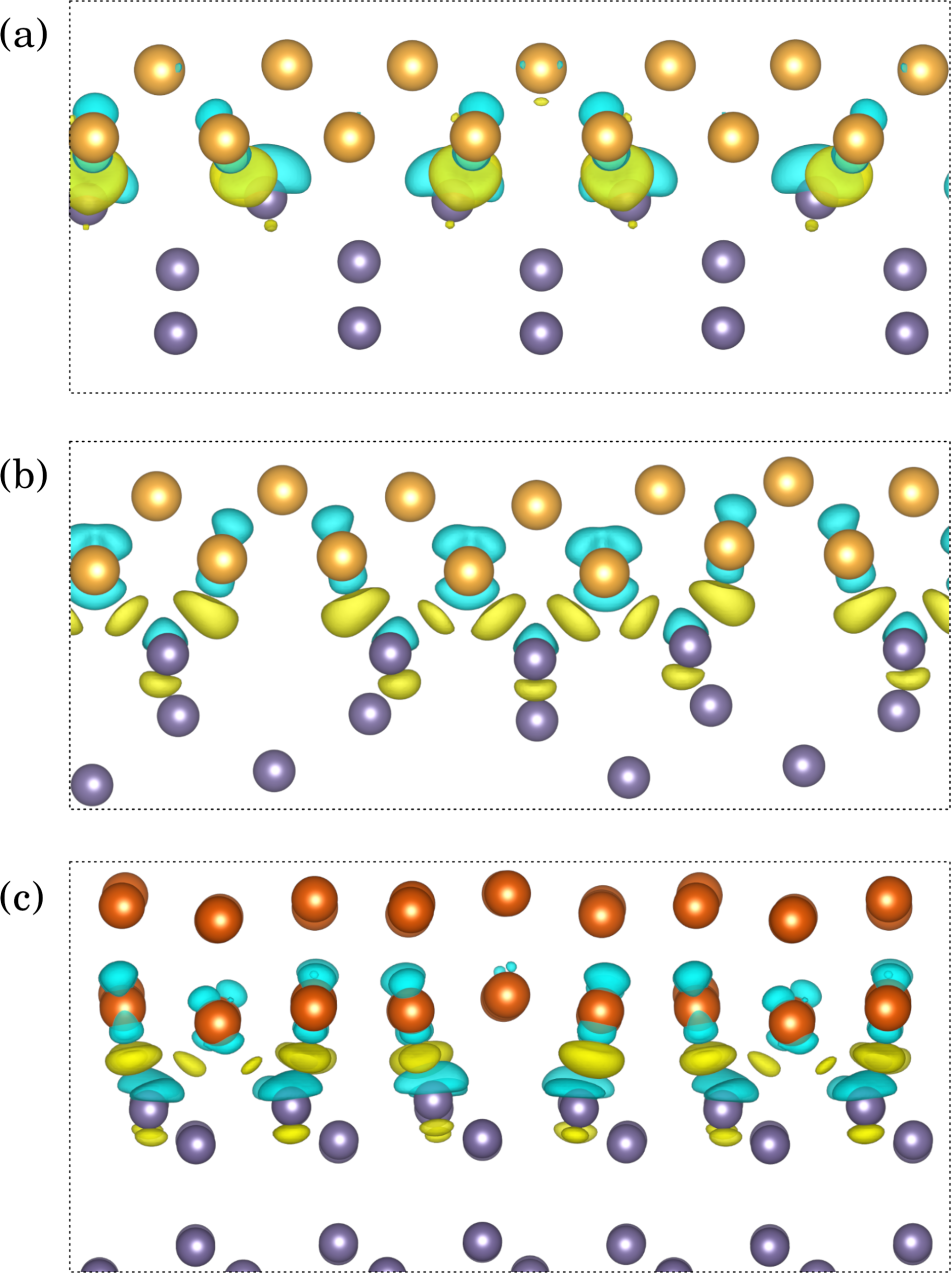
The charge density differences (0.0045 *e*/Å^−3^) for variants *C* (a) and *D* (b) of the Au-fcc(011)/Ge(001) interface compared with the isosurface of the Au-hcp(010)/Ge(111) interface (c). Yellow and cyan regions indicate the electron accumulation and depletion, respectively.

The charge density differences calculated for variants *C* and *D* of the Au-fcc(011)/Ge(001) interface and for the Au-hcp(010)/Ge(111) interface are illustrated in Figure 13 with yellow regions representing charge accumulation and light blue regions indicating charge depletion. For all heterostructures, the charge transfer from Au and Ge interfacial atoms leads to the electron accumulation at approximately half of their distance. At the presented isocharge surface value, the enhancement of charge density between interfacial Ge atoms and their nearest neighbors in the substrate is also observed for variant *D* and the Au-hcp/Ge interface.
